# Case report: Neonatal diabetes mellitus caused by *KCNJ11* mutation presenting with intracranial hemorrhage

**DOI:** 10.3389/fneur.2023.1072078

**Published:** 2023-03-03

**Authors:** Bo Wu, Wei Xu

**Affiliations:** Department of Pediatrics, Shengjing Hospital of China Medical University, Shenyang, Liaoning, China

**Keywords:** neonatal diabetes mellitus, *KCNJ11*, intracranial hemorrhage, epilepsy, encephalomalacia

## Abstract

Neonatal diabetes mellitus (NDM) is a rare type of monogenic diabetes. At present, most published studies have focused on the types of gene mutations associated with NDM and the therapeutic effect of sulfonylureas (SUs) on the disease; few studies on NDM-associated intracranial hemorrhage (ICH) exist. In addition, p.V59M mutations generally lead to intermediate DEND (iDEND: intermediate developmental delay and neonatal diabetes) syndrome without epilepsy. Here, we present a case of a 1-month-old male infant who was diagnosed with NDM caused by a *KCNJ11* missense mutation (p.V59M), presenting with cerebral injury. In the early stage of the disease, continuous insulin dose adjustment did not achieve an ideal level of blood glucose. Although blood glucose was subsequently controlled by oral SUs, which were administered after the genetic test result, the patient still displayed epilepsy and developmental delay. In this case report, we present our experience in the treatment of the infant, switching from insulin to oral SUs and we thought that SUs have limited effects on improving the prognosis of neurodevelopmental disturbances in NDM with foci of encephalomalacia. In addition, there may be a relationship between *KCNJ11* missense mutations and cerebral injury, and further research must be carried out to confirm these points.

## 1. Introduction

Neonatal diabetes mellitus (NDM), a rare genetic disease (minimal incidence: 1 in 90,000 live births) with variations in different ethnic groups, refers to a type of diabetes mellitus that develops within 6 months after birth (1). In terms of clinical prognosis, NDM can be categorized into transient NDM (TNDM) and permanent NDM (PNDM) (2). In response to glucose, the adenosine triphosphate (ATP)-dependent potassium channel (K-ATP channel) plays a critical role in stimulating insulin secretion by pancreatic β cells. Nearly half of NDM cases are caused by *KCNJ11* or *ABCC8* mutations, which impaired the ability of the ATP to bind to and block mutant K-ATP channel activity (3).

Clinical severity can range from isolated TNDM to the most severe cases affected by neurodevelopmental disability, seizures (developmental delay, epilepsy, and neonatal diabetes [DEND] syndrome), and insensitivity to sulfonylurea (SU) treatment (4). Research has revealed that, among patients with NDM caused by *KCNJ11* mutations, ~25% have DEND syndrome (5). SUs can reduce the agonistic effect of ATP on the channel by competitively binding to sulfonylurea receptor-1 (SUR1), decreasing the channel opening, and further promoting the release of insulin. The effect of SUs on NDM is significantly more efficacious than that of insulin alone, and such treatment is both safe and highly effective in the long-term (6). The proportion of patients with PNDM whose treatment was successfully switched from insulin to oral SUs has reached nearly 90% (7). In addition, SUs can have a significant effect on NDM-presenting features of neurodevelopmental disorders (8). Some studies found that diabetic ketoacidosis (DKA) was present in 78.8% of patients with mutations in *KCNJ11*/*ABCC8* genes and neurological involvement may be worsened by improper treatment (9, 10). However, only two cases of NDM associated with intracranial hemorrhage (ICH) can be found in the literature (11, 12).

In the present manuscript, we report the case of a 1-month-old infant with NDM who presented with ICH and DKA as the first clinical manifestations and whose neurological condition did not improve despite the initiation of treatment with SUs. The optimal treatment for NDM with central nervous system (CNS) features, especially in infants with intracranial lesions, remains to be further researched.

## 2. Case description

A 1-month-old male infant was admitted to the hospital with generalized tonic-clonic seizures and dyspnea for 15 h. He was a normal term infant without a history of abnormal birth (e.g., craniocerebral trauma, neonatal birth injury, hypoxia, etc.) and was fed formula milk since birth. The parents were not consanguineous; both were healthy and denied any family history of diabetes. The child's admission weight was 3 kg, which represented an increase of only 0.1 kg over a period of 1 month. Although the child had convulsions, both eyes had equal pupil size and the pupillary reaction to light was sensitive. Other symptoms at admission were as follows: somnolence, Kussmaul respiration, dry skin with poor elasticity, sunken anterior fontanelle, and cold extremities with prolonged capillary refilling time. Blood gas analysis and blood glucose examination exhibited the following values: pH 6.93, HCO_3_-3.0 mmol/L, base excess −28 mmol/L, blood glucose 33.3 mmol/L, and no signs of electrolyte disorders, suggesting the presence of severe DKA. Indeed, urine testing showed 4+ urine ketones. The fasting C-peptide (d2) and insulin levels were 0.27 ng/mL and 0.6 mU/L, respectively. Upon testing, type 1 diabetes-related autoantibodies all showed negative results.

**Figure 1:**
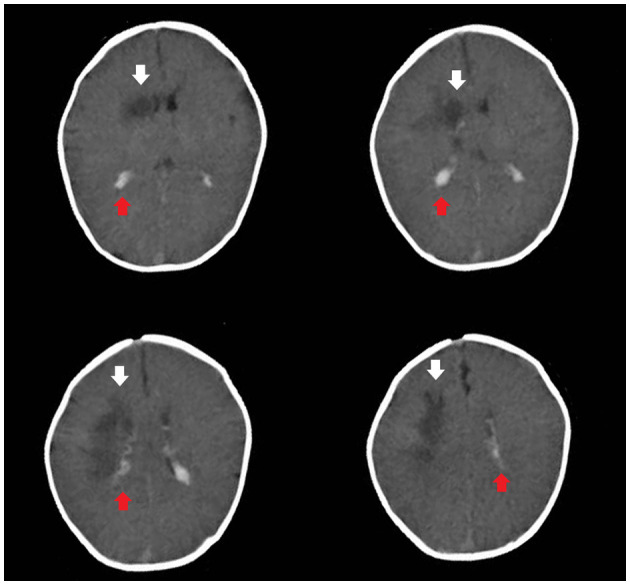
Brain computed tomography revealed subarachnoid hemorrhage (the red arrow) and irregular hypointensity shadows beside the right lateral ventricle (the white arrow).

Based on these data, the child was diagnosed with NDM. Rehydration and correction of acidosis were performed according to the consensus statement on DKA published by the International Society for Pediatric and Adolescent Diabetes (13). After 24 h, two consecutive negative ketonuria test results, a pH >7.3 and a blood glucose level < 12 mmol/L, indicated substantially corrected ketoacidosis. However, the child still presented somnolence. Brain computed tomography revealed subarachnoid hemorrhage and irregular hypointensity shadows beside the right lateral ventricle (Figure 1).

**Figure 2:**
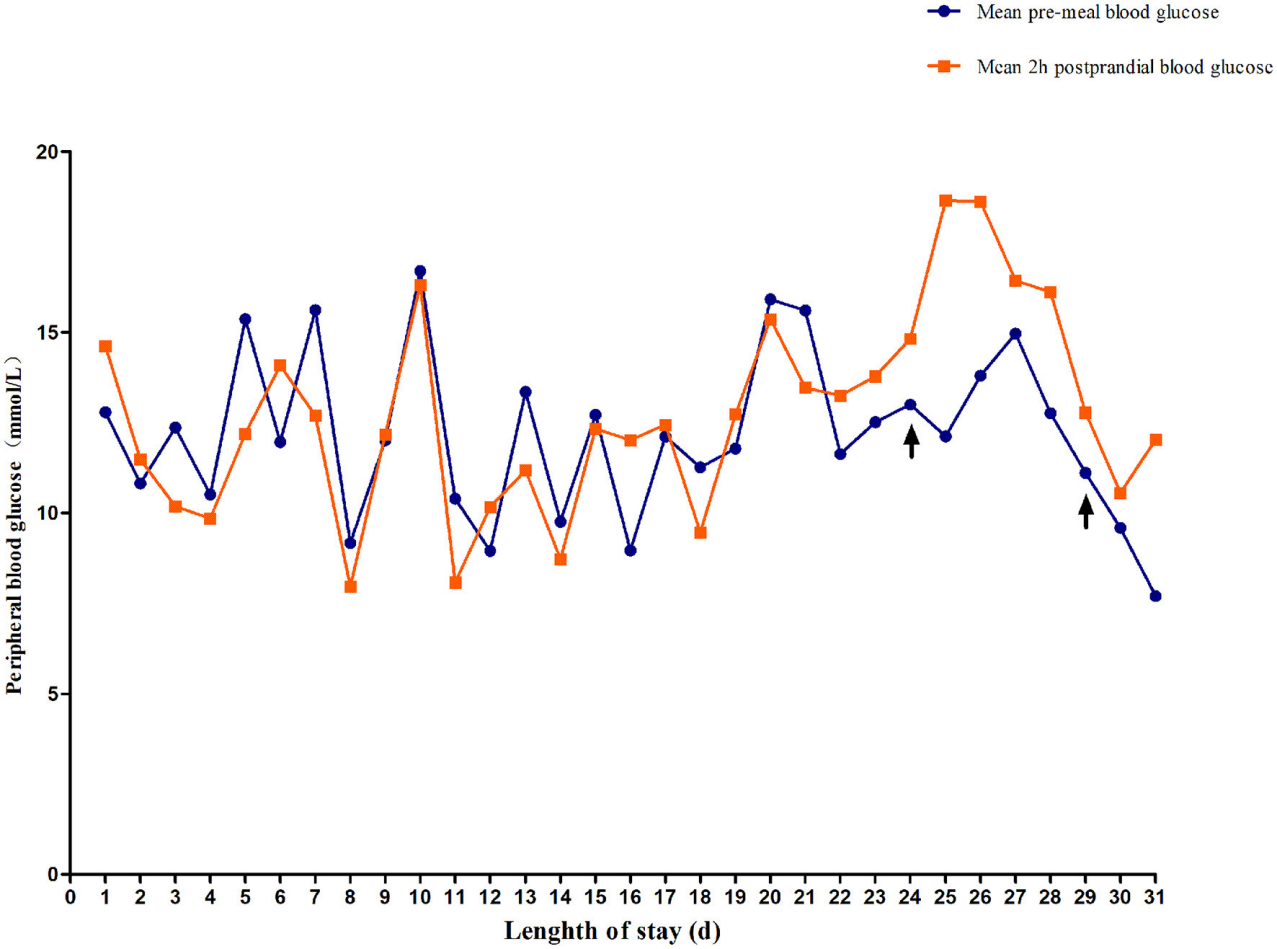
Changes in fasting blood glucose and 2-h postprandial blood glucose. On day 24, the child was treated with low-dose sulfonylureas (SUs). On day 29, blood glucose fluctuation was reduced by combined treatment with oral SUs and small and frequent meals.

The blood glucose levels were difficult to control for more than 2 weeks. Between days 3 and 18 after the admission, the child was switched to subcutaneous regular insulin injections and fed formula milk every 4 h. The patient's daily blood glucose levels fluctuated significantly and irregularly. Blood glucose was monitored a total of 276 times within a 16-day period. Preprandial blood glucose levels higher than 11.1 mmol/L and lower than 3.6 mmol/L occurred 80 (58%) and six times (4.3%), respectively. Postprandial blood glucose levels higher than 11.1 mmol/L and lower than 3.6 mmol/L occurred 78 (56.52%) and nine times (6.5%), respectively. A line graph of the blood glucose levels before and 2 h after the daily meals demonstrates that the patient's blood glucose fluctuated widely (Figure 2).

**Figure 3:**
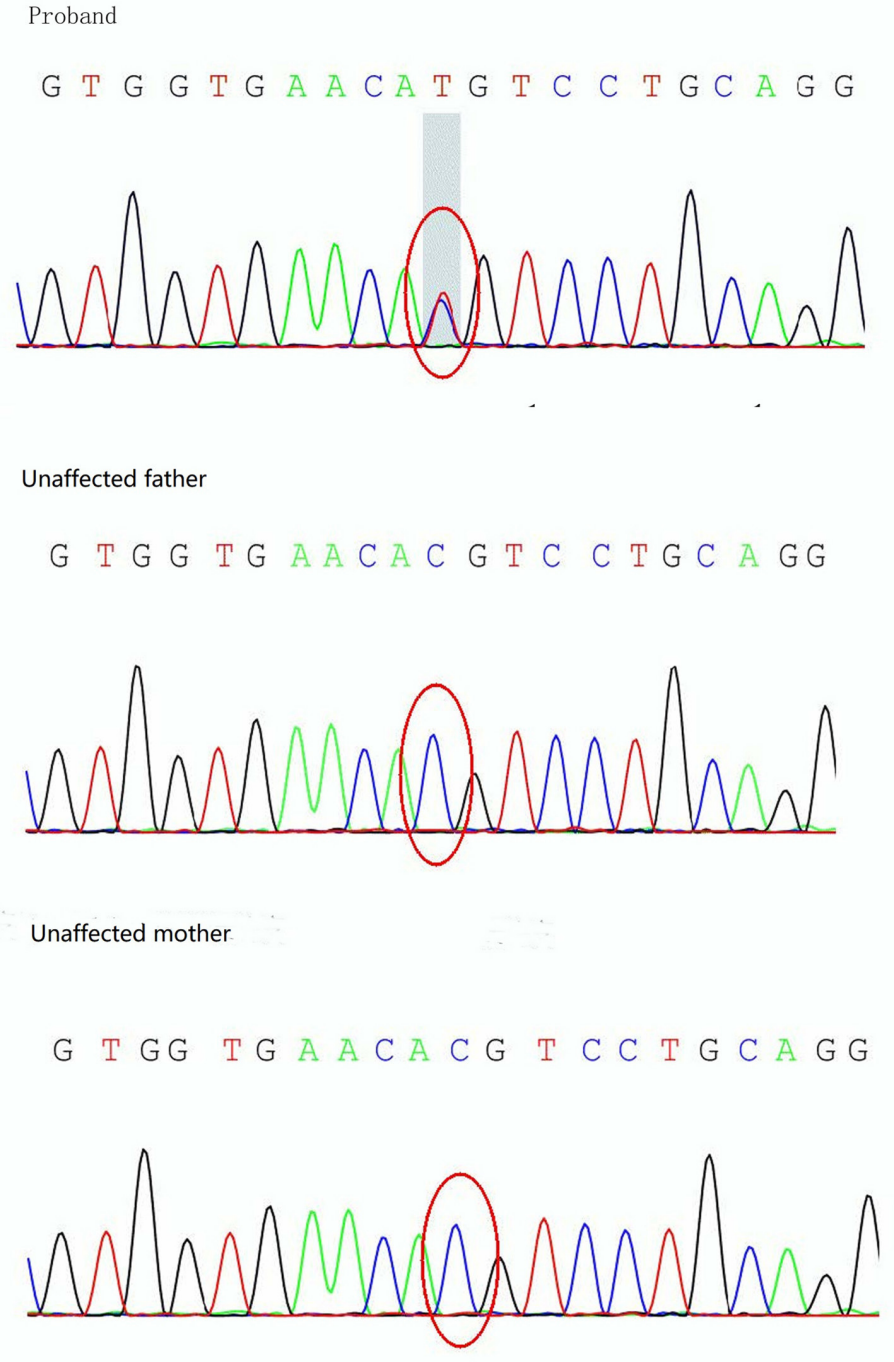
Sanger sequencing analysis of the *KCNJ11* gene showing detection of a mutation, c.175G>A (p.V59M), in the proband.

On day 19 after admission, c.175G>A heterozygous mutation in exon 1 (NM_ 000525.3) of *KCNJ11* was found by a targeted gene panel sequencing, resulting in the erroneous encoding of the amino acid at position 59 as methionine instead of valine (p.V59M) that generally leads to iDEND syndrome (Figure 3). The patient initially received small doses of oral glibenclamide (0.15 mg/kg/d q8h), which were gradually increased to 0.2 mg/kg/d q 8h. Insulin administration was tapered and fully replaced by oral SUs 8 days later. As a result, the fluctuation in blood glucose levels was reduced; however, the blood glucose levels were still high. As a potential solution, we speculated that it may have been beneficial to control his blood glucose by the frequent administration of small amounts of food. Therefore, his diet was planned as small and frequent meals (eight times a day), and glibenclamide was adjusted to 0.3 mg/kg/d and administered once every 6 h. As a result, the blood glucose levels were significantly more stable; the incidence of peripheral blood glucose levels >15 mmol/L before meals dropped to ~12% and that of blood glucose levels < 4 mmol/L 2 h after meals dropped to ~6%. Figure 2 shows that blood glucose levels tended to be stable over time. Therefore, the child was discharged after 35 days of hospitalization.

**Figure 4:**
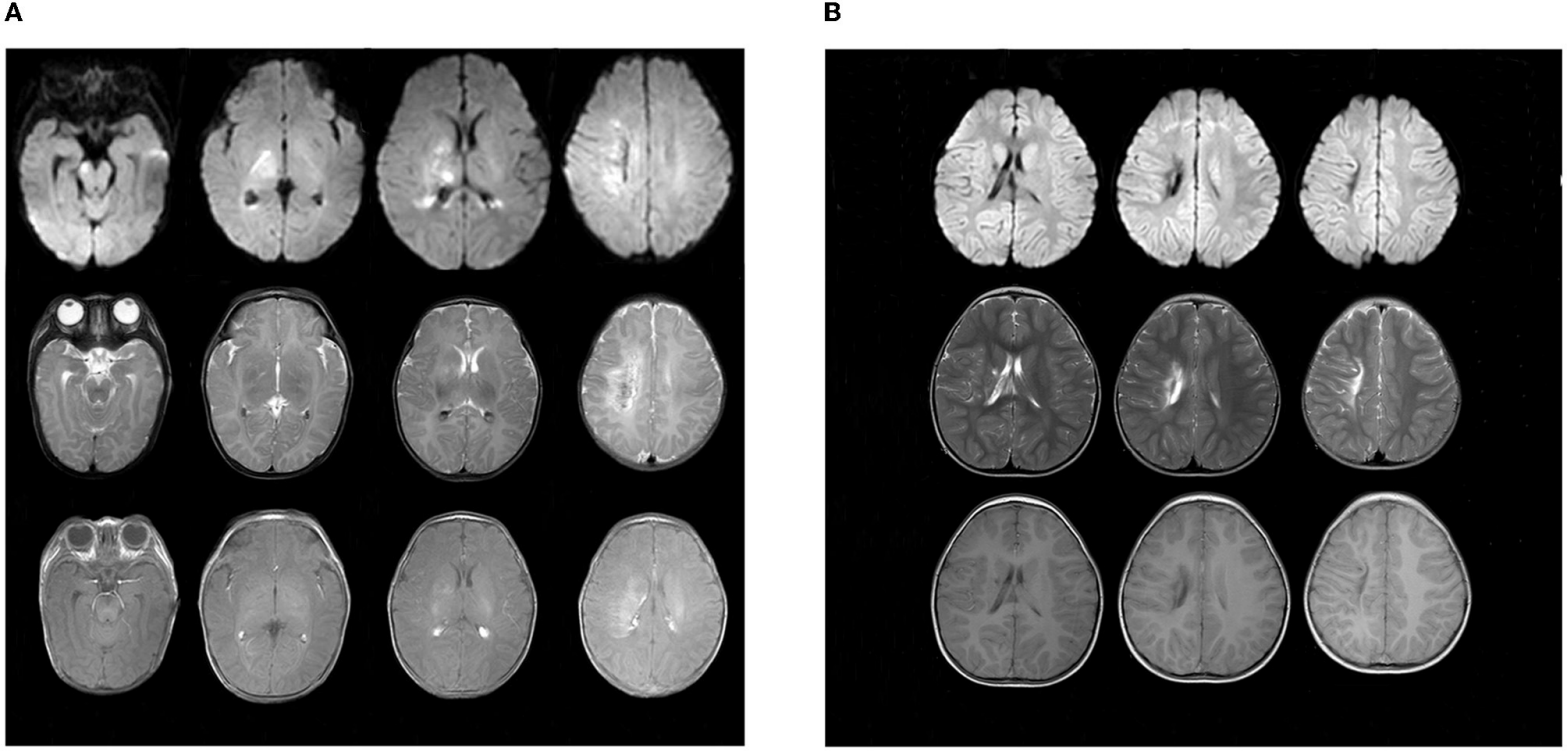
Findings in sequential MRI brain scans. **(A)** was taken upon admission in October 2018, showing multiple spots of white matter hyperintensities (in the right central semiovale, lateral ventricle, and basal ganglia) with focal hemorrhage. Patchy, limited diffuse, high signal intensity can be seen in the right central semiovale, lateral ventricle, basal ganglia, and the right side of the brain stem in diffusion-weighted imaging (DWI). **(B)** Obtained in March 2021, there were still multiple cerebral injuries with enlarged encephalomalacia in the right centrum semiovale, lateral ventricle, and basal ganglia compared with those seen in the previous scan.

The child did not have seizures during hospitalization. However, the video electroencephalogram (EEG) results showed that there were several subclinical epileptiform discharges, which were detected by the scalp electrodes located on the bilateral frontal lobes. Brain magnetic resonance imaging (MRI) was performed on day 14 after admission and showed multiple cerebral injuries with focal hemorrhage (involving the right central semiovale, lateral ventricle, basal ganglia, and right internal capsule) and bilateral intraventricular hemorrhages (Figure 4). At the age of 4 months, the infant presented with convulsions, which manifested as nodding movements, arm extension, and abnormal crying. Spasms lasted 2–6 s in rhythmic strings over 20- to 60-min periods. The patient was diagnosed with infantile spasm (IS) because of the characteristic EEG presenting patterns of hypsarrhythmia. The brain MRI showed a focal area of injury and hemorrhage that was smaller than that seen at the previous examination, as well as foci of encephalomalacia. Oral topiramate (2 mg/kg/d bis in die [BID]) was administered as an anti-epileptic treatment.

At present, the child is 2 years old, his blood glucose is well controlled by oral crushed glibenclamide (0.11 mg/kg/d q8h), and his HbA1c is 6.8%. However, his epilepsy is poorly controlled by oral topiramate (4.5 mg/kg/d BID). Enlarged brain injuries and encephalomalacia were observed in the right central semioval, lateral ventricle, and basic ganglia (Figure 4). Furthermore, he is only able to say “dad” and “mother” and cannot walk by himself due to left hemiplegia.

## 3. Discussion

This article reported a case of NDM caused by a p.V59M mutation in the *KCNJ11* gene, presenting as DKA, ICH, and encephalomalacia in an infant whose blood glucose was difficult to control *via* insulin treatment. In the end, blood glucose was stabilized by switching treatment to oral SUs and changing the feeding mode. However, epilepsy and developmental retardation accompanied by encephalomalacia were found during follow-up.

We used “V59M” as the keyword for literature retrieval and reviewed this literature (Table 1). It is generally believed that NDM caused by p.V59M and p.V59A mutations usually leads to iDEND syndrome without epilepsy, and presents a better clinical response to SUs compared with NDM caused by p.V59G mutations (14). However, our patient with p.V59M mutation had complications of ICH and encephalomalacia, and his neurological condition, referring to epilepsy and developmental retardation, did not improve despite the early treatment with SUs. Although there have been some cases of p.V59M mutations with reported seizures at some point in life, these do not typically occur in the neonatal period (15–17). In addition, one study finds that there are no significant correlations between specific neurodevelopmental impairments and genotype in the subgroup of patients with mutations in K-ATP channel subunit genes (18). In patients with NDM, cerebral MRI usually shows normal brain anatomy. Only some patients have presented with multiple punctate white matter hyperintensities on T2 and fluid-attenuated inversion recovery (FLAIR) sequences (17, 19, 20). Ja Hyang Cho et al. reported the case of a child diagnosed with NDM caused by p.R201H mutation, whose brain MRI demonstrated a symmetric high signal intensity of periventricular white matter on T2-weighted and FLAIR images at 21 months of age (21). In comparison, our patient presented with similar multiple white matter lesions but differed due to the presence of local hemorrhage and a larger asymmetric brain defect as well as the age at onset.

**Table 1:** Clinical data of patients with heterozygous p.V59M mutation.

**Age at diagnosis (months)**	**Age SUs switch (months)**	**DKA at presentation**	**Abnormal brain anatomy**	**Epilepsy**	**iDEND syndrome**	**HbA1c after SUs treatment(%)**	**Improvement in neurological features**
**Svalastoga et al**. **(****17****)**							
2	5	+	–	–	+	7.2	NA
4	15	+	–	+	+	6.4	NA
1	14	+	–	–	+	5.7	NA
4	7	+	+	–	+	5.7	NA
2	4	+	–	–	+	6.4	NA
**Slingerland et al**. **(****15****)**							
0.06	132	+	NA	+	+	6.5	+
**Li et al**. **(****16****)**							
120d	120d	–	–	–	+	NA	NA
180d	5y4m	+	–	+	+	NA	+
**Slingerland et al**. **(****40****)**							
1	23	+	–	+	+	6.5	+
**Sang et al**. **(****44****)**							
2.5	NA	+	NA	–	+	NA	NA
**Hashimoto et al**. **(****35****)**							
2	6	+	NA	–	+	6.5	+
7	30	NA	NA	–	+	8.0	NA
**Gloyn et al**. **(****45****)**							
1.5	NA	+	NA	–	+	6.8	–
**Mohamadi et al**. **(****46****)**							
11.5	183	–	NA	–	+	9.9	NA
**Vaxillaire et al**. **(****20****)**							
4	NA	+	+	NA	+	NA	NA
**Ting et al**. **(****47****)**							
1	72	+	–	–	+	7.25	NA
4.5	180	+	NA	–	+	7.7	NA
**Kim et al**. **(****48****)**							
1.5	45	+	NA	–	+	5.8	NA
**Nieves-Rivera et al**. **(****36****)**							
1	12	+	NA	–	+	5.8	+

SUs, sulfonylureas; DKA, diabetes ketoacidosis; iDEND syndrome, intermediate developmental delay and neonatal diabetes without epilepsy; NA, data not available.

Children who develop DKA are at risk for intracranial vascular complications including vasogenic edema, hemorrhage, and stroke (22). Among them, cerebral edema (CE) is a potentially devastating complication of DKA in children with a morbidity of 0.7%-1% which is higher in the at-risk groups (23). Encephalomalacia in our case may be caused by prolonged severe CE. The pathophysiologic mechanisms of CE are complicated and can be roughly divided into three categories: cytotoxic, vasogenic, and osmotic (24). In addition, oxidative stress and ischemia-reperfusion injury may be involved in brain injury (25, 26). Signs and symptoms of clinically apparent CE usually become evident within the first 12 h of treatment but can occur before treatment has been initiated (24). In addition, diabetes as a condition of accelerated vascular aging is an important risk factor for cerebral injury. Diabetes also mitigates the recovery following ICH (27). However, it is unusual that our patient presented with extensive encephalomalacia during the acute phase.

Is there a relationship between *KCNJ11* missense mutations and cerebral injury? A review of the relevant literature revealed only two cases of ICH; one case was reported in 1999 and the other was published in a Chinese magazine. Unfortunately, the previous case reports did not perform genetic testing (11, 12). In one of the studies, the infant died on the second day of admission, while the other neurological condition was unknown because of loss of follow-up. The gene *KCNJ11* encodes Kir6.2 a major subunit of the K-ATP channels expressed in a variety of cell types in the brain. The mutation caused by p.V59M can disrupt the organization and impairs the maturation of cortical neural networks (28). Patients with the p.V59M genotype show severe affection for cognitive abilities, collectively suffering from a moderate intellectual disability (17). One study found that some CNS features are not present in individuals with insulin gene mutations that are the second genetic cause of PNDM, which indicates that they occur not as a result of the lifelong metabolic disturbance caused by PNDM but, rather, as a consequence of the impaired K-ATP channel function in the brain (29). Cortical neurons lacking the Kir6.2 gene are more vulnerable to ischemic insults than wild-type neurons (30). By using human stem cell-derived cerebral organoids it was found that the *KCNJ11* mutation (p.V59M) disrupts organization and impairs the maturation of cortical neural networks (28). In vascular smooth muscle cells, K-ATP channels opening determines a hyperpolarized state that leads to vessel dilation (31). Therefore, we speculate that *KCNJ11* mutation plays a major role in NDM with cerebral injury and DKA creates conditions or accelerates the occurrence. However, this is only the third report of NDM associated with ICH but highlights the need for further research on brain lesions in NDM.

The origin of EEG abnormalities was inconsistent with the location of brain injuries in our patient. In fact, many lesions are not intrinsically epileptogenic but induce seizures by generating reactions in the surrounding brain tissue with which they are in contact (32). Some lesions may induce microchanges in the brain tissue located at a significant distance from the epileptogenic lesion visible on MRI (32). Scalp EEG only reveals a small percentage of the interictal epileptiform discharges detected by depth or subdural electrodes; furthermore, the insular and mesio-basal regions of the brain are not covered by surface EEG (33). Therefore, scalp EEG presents a relatively low spatial resolution, which could be improved with the use of intracranial EEG. Another potential explanation is related to the feedback regulation and sensitivity of MRI in detecting the entire lesion (34).

It is generally believed that many aspects of cognitive function are improved by SUs treatment (15, 35, 36). However, there is no abnormal brain anatomy in these patients. In addition, the improvement is observed in the follow-up for more than 1 year after changing to SUs treatment, which could not exclude the results of rehabilitation training. Some cases in a family had a severe clinical presentation and more severe outcome than those of others presenting the same mutation, suggesting that long-term neurological morbidity can occur independently of the underlying genetic mutation (37). Other studies suggest that SUs only have a subtherapeutic concentration in the cerebrospinal fluid due to their poor ability to penetrate the blood brain barrier, and they do not affect the overexpression of SUR1 in the forebrain, which results in seizure resistance (30, 38). In addition, SUs improve long-term cognitive deficits following ICH by the Sur1-Trpm4 channel but not the Sur1-KATP channel, and the neurological actions of SUs are initially principally on peripheral (nerve or muscle) rather than on central (brain) K-ATP channels (39, 40). Therefore, the same mutant genotype has differences in phenotype and response to treatment because of the difference in individual and SUs dosage. Moreover, SUs may have limited effects on improving the prognosis of neurodevelopmental disturbances in NDM with foci of encephalomalacia.

Should we use SUs earlier to control blood glucose, especially for infants? It is generally recommended to initiate SUs treatment in a patient with NDM once their genetic test has confirmed the presence of activating mutations of the K-ATP channel (37). In addition, some studies suggest that patients who are highly suspected to have NDM could receive a trial treatment with SUs even without performing genetic testing (16, 41, 42). Patients who were successfully switched from insulin to SUs treatment certainly had an improvement in HbA1c levels (43). The use of an insulin pump may be an ideal choice for our patient in the early administration; however, it is difficult to use due to the low weight and thin subcutaneous fat in infants. Therefore, SUs treatment combined with dietary changes is a better choice for infants with NDM to control blood glucose.

In conclusion, the findings of this case suggest that patients with *KCNJ11* gene missense mutations may be more prone to cerebral injury. This case report expands on the cause of acute cerebral injuries such as ICH and encephalomalacia in infants. Furthermore, SUs were shown to have limited effects on improving the prognosis of neurodevelopmental disturbances in NDM with foci of encephalomalacia. Frequent administration of small amounts of food is a better choice for infants with NDM to control blood glucose.

## Data availability statement

The datasets presented in this article are not readily available because of ethical and privacy restrictions. Requests to access the datasets should be directed to the corresponding author.

## Ethics statement

Written informed consent was obtained from the minor(s)' legal guardian/next of kin for the publication of any potentially identifiable images or data included in this article.

## Author contributions

BW and WX designed and performed the research, analyzed and interpreted the data, and wrote the manuscript. All authors have read and approved the manuscript.
